# Bis[[(6-carb­oxy­pyridazine-3-carboxyl­ato-κ^2^ *N* ^2^,*O* ^3^)lithium]-μ-penta­hydrogen­dioxy­gen(1+)]

**DOI:** 10.1107/S1600536810039176

**Published:** 2010-10-09

**Authors:** Wojciech Starosta, Janusz Leciejewicz

**Affiliations:** a Institute of Nuclear Chemistry and Technology, ul. Dorodna 16, 03-195 Warszawa, Poland

## Abstract

The structure of the title compound, [Li(C_6_H_3_N_2_O_4_)_2_(H_5_O_2_)], is composed of centrosymmetric monomers in which an Li^I^ ion is chelated by two *N*,*O*-bonding groups donated by two ligands. The Li^I^ ion and both ligand mol­ecules are coplanar [r.m.s. deviation 0.0047 (2) Å] and water O atoms are in the axial positions. The second carboxyl group of each ligand remains protonated. An additional H atom, located between adjacent coordinated water mol­ecules and observed on Fourier maps, maintains the charge balance within the monomers and bridges them by short symmetric hydrogen bonds of 2.518 (3) Å to form catenated ribbons. The monomers also inter­act *via* hydrogen bonds in which water and carboxyl O atoms act as donors.

## Related literature

For the crystal structures of 3*d* metal complexes with pyrid­azine-3,6-dicarboxyl­ate and water ligands, see: El Gueddi *et al.* (1996 ▶); Escuer *et al.* (1997 ▶); Gryz *et al.* (2006 ▶); Sun *et al.* (2007 ▶, 2008 ▶). For the structures of complexes with Mg^II^, see: Gryz *et al.* (2004 ▶). For the structures of complexes with Pb^II^, see: Sobanska *et al.* (1999 ▶). For the structures of both modifications of pyridazine-3,6-dicarb­oxy­lic acid, see: Suecur *et al.* (1987 ▶); Starosta & Leciejewicz (2004 ▶).
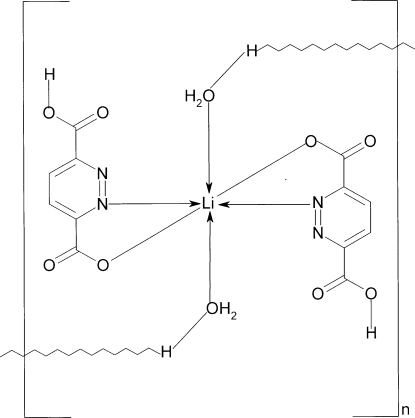


## Experimental

### 

#### Crystal data

[Li(C_6_H_3_N_2_O_4_)_2_(H_5_O_2_)]
*M* *_r_* = 378.19
Monoclinic, 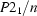

*a* = 4.903 (1) Å
*b* = 24.640 (5) Å
*c* = 6.6020 (13) Å
β = 111.60 (3)°
*V* = 741.6 (3) Å^3^
*Z* = 2
Mo *K*α radiation
μ = 0.15 mm^−1^
*T* = 295 K
0.42 × 0.39 × 0.07 mm

#### Data collection

Kuma KM-4 four-circle diffractometer
Absorption correction: analytical (*CrysAlis RED*; Oxford Diffraction, 2008 ▶) *T*_min_ = 0.961, *T*_max_ = 0.999
4355 measured reflections
2181 independent reflections
1207 reflections with *I* > 2σ(*I*)
*R*_int_ = 0.160
3 standard reflections every 200 reflections  intensity decay: 0.8%

#### Refinement

*R*[*F* ^2^ > 2σ(*F* ^2^)] = 0.047
*wR*(*F* ^2^) = 0.117
*S* = 1.01
2181 reflections
135 parameters
3 restraints
H atoms treated by a mixture of independent and constrained refinement
Δρ_max_ = 0.40 e Å^−3^
Δρ_min_ = −0.31 e Å^−3^

### 

Data collection: *KM-4* (Kuma, 1996 ▶); cell refinement: *KM-4*; data reduction: *DATAPROC* (Kuma, 2001 ▶); program(s) used to solve structure: *SHELXS97* (Sheldrick, 2008 ▶); program(s) used to refine structure: *SHELXL97* (Sheldrick, 2008 ▶); molecular graphics: *SHELXTL* (Sheldrick, 2008 ▶); software used to prepare material for publication: *SHELXTL*.

## Supplementary Material

Crystal structure: contains datablocks I, global. DOI: 10.1107/S1600536810039176/rk2232sup1.cif

Structure factors: contains datablocks I. DOI: 10.1107/S1600536810039176/rk2232Isup2.hkl

Additional supplementary materials:  crystallographic information; 3D view; checkCIF report

## Figures and Tables

**Table 1:** Hydrogen-bond geometry (Å, °)

*D*—H⋯*A*	*D*—H	H⋯*A*	*D*⋯*A*	*D*—H⋯*A*
O6—H63⋯O6^i^	1.26	1.26	2.518 (3)	180
O6—H61⋯O1^ii^	0.84 (2)	1.82 (2)	2.608 (2)	157 (3)
O3—H31⋯O2^iii^	0.96 (4)	1.56 (4)	2.525 (2)	176 (2)
O6—H62⋯O3^iv^	0.82 (2)	2.42 (2)	2.9957 (19)	128 (3)
O6—H62⋯N2^iv^	0.82 (2)	1.93 (2)	2.712 (2)	159 (3)

Symmetry codes: (i) 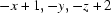; (ii) 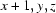; (iii) 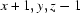; (iv) 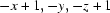.

**Fig. 1.**
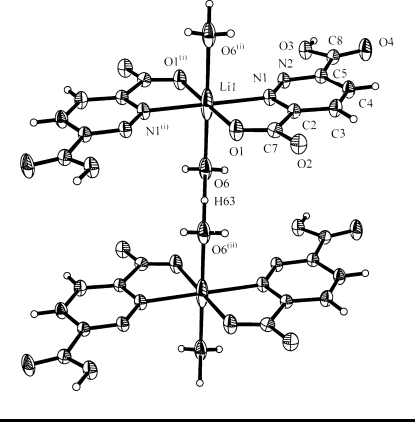
A structural unit of title compound with atom labelling scheme. Displacement ellipsoids are drawn at 50% probability level. Symmetry codes: (i) -x, -y, -z + 1; (ii) -x + 1, -y, -z + 2.

**Fig. 2.**
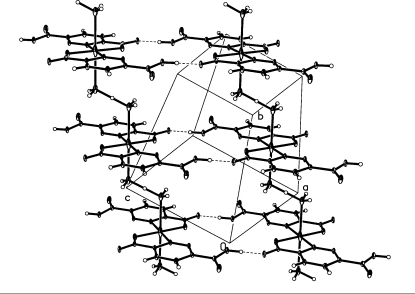
Packing diagram of the structure.

## supplementary crystallographic information

### Comment

Studies of 3d metal complexes with pyridazine-3,6-dicarboxylate and water ligands revealed a variety of structures: from a monomeric anion in an ionic complex [Mg{C_6_H_2_N_2_O_4_)_2_(H_2_O)_2_}]^2-^(N_2_H_6_)^2+^ (Gryz *et al.*, 2004) and dimeric molecules as in Ni, Co and Zn complexes (Escuer *et al.*, 1997; Gryz *et al.*, 2006; Sun *et al.*, 2008) to coordination polymers as in Mn^II^ complexes (El Gueddi *et al.*, 1996; Sun *et al.*, 2007, 2008). The structure of a Pb^II^ complex shows also a polymeric pattern (Sobanska *et al.*, 1999). The structure of the title compound is composed of monomers in which a Li^I^ ion located in a centre of symmetry is chelated by two *N*,*O* bonding groups donated by two symmetry related ligand molecules and by two symmetry related aqua O atoms in axial positions. The coordination is slightly distorted octahedral. The ligand molecules and a Li^I^ ion are coplanar [r.m.s. 0.0047 (2) Å]. The second carboxylic group of each ligand remains protonated and makes an angle of 5.9 (1)° with the pyridazine plane. Bond lengths and angles within the ligand ring are close to those reported earlier for both structures of the parent acid (Suecur *et al.*, 1987; Starosta & Leciejewicz, 2004). An additional proton in a special position located between coordinated water molecules is clearly observed on Fourier maps. It maintains the charge balance within monomers and bridges them by short symmetric hydrogen bonds of 2.518 (3) Å with O6—H63—O6^(ii)^ angle of 180° to form catenated ribbons. Symmetry code: (ii) -*x* + 1, -*y*, -*z* + 2. The latter are held together *via* hydrogen bonds in which water and protonated carboxylate O atoms act as donors and carboxylate O atoms and hetero-N atoms in adjacent ribbons as acceptors.

### Experimental

The title compound was synthesized by mixing of boiling aqueous solutions, one containing 1 mmol of pyridazine-3,6-dicarboxylic acid, the other - 1 mmol of lithium hydroxide (Aldrich). The mixture was boiled under reflux for 3 h and after cooling to room temperature, filtered and left to crystallize. Few days later, colourless single crystals in the form of thin plates were found after evaporation to dryness. They were extracted, washed with cold ethanol and dried in the air.

### Refinement

Water H atoms were located in a difference map and were allowed to ride on the parent atom with *U*_iso_(H) = 1.5*U*_eq_(O). H atoms attached to pyridazine-ring C atoms were located at calculated positions and treated as riding on the parent atoms, with C—H = 0.93 Å and *U*_iso_(H) = 1.2*U*_eq_(C).

### Crystal data

**Table:**

[Li(C_6_H_3_N_2_O_4_)_2_(H_5_O_2_)]	*F*(000) = 388
*M**_r_* = 378.19	*D*_x_ = 1.694 Mg m^−^^3^
Monoclinic, *P*2_1_/*n*	Mo *K*α radiation, λ = 0.71073 Å
*a* = 4.903 (1) Å	Cell parameters from 25 reflections
*b* = 24.640 (5) Å	θ = 6–15°
*c* = 6.6020 (13) Å	µ = 0.15 mm^−^^1^
β = 111.60 (3)°	*T* = 295 K
*V* = 741.6 (3) Å^3^	Plate, colourless
*Z* = 2	0.42 × 0.39 × 0.07 mm

### Data collection

**Table:**

Kuma KM-4 four-circle diffractometer	1207 reflections with *I* > 2σ(*I*)
Radiation source: fine-focus sealed tube	*R*_int_ = 0.160
graphite	θ_max_ = 30.1°, θ_min_ = 1.7°
profile data from ω/2θ–scans	*h* = −6→6
Absorption correction: analytical (*CrysAlis RED*; Oxford Diffraction, 2008)	*k* = 0→34
*T*_min_ = 0.961, *T*_max_ = 0.999	*l* = −9→9
4355 measured reflections	3 standard reflections every 200 reflections
2181 independent reflections	intensity decay: 0.8%

### Refinement

**Table:**

Refinement on *F*^2^	Primary atom site location: structure-invariant direct methods
Least-squares matrix: full	Secondary atom site location: difference Fourier map
*R*[*F*^2^ > 2σ(*F*^2^)] = 0.047	Hydrogen site location: inferred from neighbouring sites
*wR*(*F*^2^) = 0.117	H atoms treated by a mixture of independent and constrained refinement
*S* = 1.01	*w* = 1/[σ^2^(*F*_o_^2^) + (0.0251*P*)^2^ + 0.0008*P*] where *P* = (*F*_o_^2^ + 2*F*_c_^2^)/3
2181 reflections	(Δ/σ)_max_ < 0.001
135 parameters	Δρ_max_ = 0.40 e Å^−^^3^
3 restraints	Δρ_min_ = −0.31 e Å^−^^3^

### Special details

**Table:**

Geometry. All s.u.'s (except the s.u. in the dihedral angle between two l.s. planes) are estimated using the full covariance matrix. The cell s.u.'s are taken into account individually in the estimation of s.u.'s in distances, angles and torsion angles; correlations between s.u.'s in cell parameters are only used when they are defined by crystal symmetry. An approximate (isotropic) treatment of cell s.u.'s is used for estimating s.u.'s involving l.s. planes.
Refinement. Refinement of *F*^2^ against ALL reflections. The weighted *R*-factor *wR* and goodness of fit *S* are based on *F*^2^, conventional *R*-factors *R* are based on *F*, with *F* set to zero for negative *F*^2^. The threshold expression of *F*^2^ > σ(*F*^2^) is used only for calculating *R*-factors(gt) *etc*. and is not relevant to the choice of reflections for refinement. *R*-factors based on *F*^2^ are statistically about twice as large as those based on *F*, and *R*-factors based on ALL data will be even larger.

### Fractional atomic coordinates and isotropic or equivalent isotropic displacement parameters (Å^2^)

**Table:**

	*x*	*y*	*z*	*U*_iso_*/*U*_eq_	
N1	0.1189 (4)	0.08134 (5)	0.4137 (2)	0.0216 (3)	
O1	−0.1491 (4)	0.04967 (4)	0.6760 (2)	0.0308 (3)	
O3	0.5111 (3)	0.11620 (5)	0.0040 (2)	0.0288 (3)	
O2	−0.2209 (3)	0.13593 (5)	0.7509 (2)	0.0297 (3)	
C5	0.2683 (4)	0.14735 (6)	0.2292 (3)	0.0208 (4)	
N2	0.2452 (4)	0.09550 (5)	0.2750 (2)	0.0215 (3)	
C2	0.0209 (4)	0.11977 (6)	0.5110 (2)	0.0196 (3)	
C7	−0.1279 (4)	0.10016 (6)	0.6606 (2)	0.0208 (4)	
C8	0.4088 (4)	0.15946 (6)	0.0675 (3)	0.0226 (4)	
O4	0.4208 (4)	0.20499 (4)	0.0057 (2)	0.0405 (4)	
C4	0.1727 (5)	0.18911 (6)	0.3287 (3)	0.0264 (4)	
H4	0.1930	0.2253	0.2965	0.032*	
C3	0.0479 (5)	0.17513 (6)	0.4753 (3)	0.0253 (4)	
H3	−0.0165	0.2014	0.5486	0.030*	
Li1	0.0000	0.0000	0.5000	0.0588 (19)	
O6	0.4949 (4)	−0.00562 (5)	0.8091 (2)	0.0415 (4)	
H61	0.606 (6)	0.0181 (8)	0.792 (4)	0.062*	
H62	0.558 (6)	−0.0364 (7)	0.807 (4)	0.062*	
H31	0.617 (7)	0.1250 (8)	−0.089 (4)	0.042 (7)*	
H63	0.5000	0.0000	1.0000	0.080 (14)*	

### Atomic displacement parameters (Å^2^)

**Table:**

	*U*^11^	*U*^22^	*U*^33^	*U*^12^	*U*^13^	*U*^23^
N1	0.0282 (8)	0.0198 (6)	0.0270 (7)	0.0007 (5)	0.0221 (6)	0.0017 (5)
O1	0.0433 (9)	0.0227 (5)	0.0420 (7)	−0.0012 (5)	0.0339 (6)	0.0023 (5)
O3	0.0415 (9)	0.0237 (5)	0.0362 (7)	0.0014 (5)	0.0319 (6)	0.0020 (5)
O2	0.0401 (9)	0.0289 (6)	0.0350 (7)	0.0004 (5)	0.0311 (5)	−0.0016 (4)
C5	0.0247 (9)	0.0204 (6)	0.0235 (7)	−0.0007 (7)	0.0160 (6)	0.0004 (5)
N2	0.0295 (9)	0.0200 (5)	0.0245 (7)	0.0004 (6)	0.0211 (6)	0.0018 (5)
C2	0.0234 (9)	0.0205 (6)	0.0215 (7)	−0.0009 (6)	0.0162 (6)	0.0000 (5)
C7	0.0215 (9)	0.0260 (7)	0.0221 (7)	−0.0010 (6)	0.0162 (6)	0.0000 (5)
C8	0.0289 (10)	0.0216 (7)	0.0243 (7)	−0.0016 (6)	0.0182 (7)	−0.0013 (5)
O4	0.0689 (11)	0.0230 (6)	0.0506 (8)	−0.0029 (7)	0.0465 (7)	0.0046 (5)
C4	0.0384 (12)	0.0187 (6)	0.0308 (9)	0.0008 (7)	0.0229 (8)	0.0013 (6)
C3	0.0352 (11)	0.0195 (7)	0.0312 (9)	0.0001 (7)	0.0239 (7)	−0.0029 (6)
Li1	0.108 (6)	0.0194 (19)	0.095 (4)	−0.006 (3)	0.091 (4)	−0.001 (2)
O6	0.0709 (13)	0.0209 (6)	0.0542 (9)	−0.0033 (6)	0.0484 (8)	0.0009 (5)

### Geometric parameters (Å, °)

**Table:**

N1—N2	1.327 (2)	C2—C7	1.507 (3)
N1—C2	1.330 (2)	C8—O4	1.2024 (19)
N1—Li1	2.2194 (14)	C4—C3	1.366 (3)
O1—C7	1.2558 (18)	C4—H4	0.9300
O1—Li1	2.0019 (15)	C3—H3	0.9300
O3—C8	1.311 (2)	Li1—O1^i^	2.0020 (15)
O3—H31	0.96 (4)	Li1—N1^i^	2.2195 (14)
O2—C7	1.241 (2)	Li1—O6	2.535 (2)
C5—N2	1.3274 (19)	Li1—O6^i^	2.535 (2)
C5—C4	1.392 (2)	O6—H61	0.836 (18)
C5—C8	1.498 (3)	O6—H62	0.822 (16)
C2—C3	1.399 (2)	O6—H63	1.2600
			
N2—N1—C2	119.32 (13)	C2—C3—H3	121.3
N2—N1—Li1	130.38 (11)	O1—Li1—O1^i^	180.0
C2—N1—Li1	110.08 (12)	O1—Li1—N1	77.50 (6)
C7—O1—Li1	119.98 (14)	O1^i^—Li1—N1	102.50 (6)
C8—O3—H31	112.3 (14)	O1—Li1—N1^i^	102.50 (6)
N2—C5—C4	122.17 (19)	O1^i^—Li1—N1^i^	77.50 (6)
N2—C5—C8	117.00 (16)	N1—Li1—N1^i^	180.0
C4—C5—C8	120.82 (14)	O1—Li1—O6	90.68 (6)
C5—N2—N1	120.70 (16)	O1^i^—Li1—O6	89.32 (6)
N1—C2—C3	122.66 (18)	N1—Li1—O6	89.54 (5)
N1—C2—C7	115.88 (13)	N1^i^—Li1—O6	90.46 (5)
C3—C2—C7	121.45 (17)	O1—Li1—O6^i^	89.32 (6)
O2—C7—O1	127.48 (19)	O1^i^—Li1—O6^i^	90.68 (6)
O2—C7—C2	116.05 (14)	N1—Li1—O6^i^	90.46 (5)
O1—C7—C2	116.45 (16)	N1^i^—Li1—O6^i^	89.54 (5)
O4—C8—O3	125.3 (2)	O6—Li1—O6^i^	180.0
O4—C8—C5	121.33 (18)	Li1—O6—H61	109.5 (17)
O3—C8—C5	113.34 (14)	Li1—O6—H62	106.8 (17)
C3—C4—C5	117.69 (15)	H61—O6—H62	112 (3)
C3—C4—H4	121.2	Li1—O6—H63	117.00
C5—C4—H4	121.2	H61—O6—H63	107.00
C4—C3—C2	117.40 (17)	H62—O6—H63	104.00
C4—C3—H3	121.3		

Symmetry codes: (i) −*x*, −*y*, −*z*+1.

### Hydrogen-bond geometry (Å, °)

**Table:**

*D*—H···*A*	*D*—H	H···*A*	*D*···*A*	*D*—H···*A*
O6—H63···O6^ii^	1.26	1.26	2.518 (3)	180
O6—H61···O1^iii^	0.84 (2)	1.82 (2)	2.608 (2)	157 (3)
O3—H31···O2^iv^	0.96 (4)	1.56 (4)	2.525 (2)	176 (2)
O6—H62···O3^v^	0.82 (2)	2.42 (2)	2.9957 (19)	128 (3)
O6—H62···N2^v^	0.82 (2)	1.93 (2)	2.712 (2)	159 (3)

Symmetry codes: (ii) −*x*+1, −*y*, −*z*+2; (iii) *x*+1, *y*, *z*; (iv) *x*+1, *y*, *z*−1; (v) −*x*+1, −*y*, −*z*+1.
